# A comparative study of the efficacy of ultrasonics and extracorporeal shock wave in the treatment of tennis elbow: a meta-analysis of randomized controlled trials

**DOI:** 10.1186/s13018-019-1290-y

**Published:** 2019-08-06

**Authors:** Chenchen Yan, Yuan Xiong, Lang Chen, Yori Endo, Liangcong Hu, Mengfei Liu, Jing Liu, Hang Xue, Abudula Abududilibaier, Bobin Mi, Guohui Liu

**Affiliations:** 1 Department of Orthopedics, Union Hospital, Tongji Medical College, Huazhong University of Science and Technology, Jiefang Rd. 1277#, Wuhan, 430022 Hubei China; 2 Department of Plastic Surgery, Brigham and Women’s Hospital, Harvard Medical School, Boston, 02152 USA

## Abstract

**Background:**

Tennis elbow or lateral epicondylitis is a common source of pain among craftsmen. Although it cannot be completely resolved, extracorporeal shock wave therapy (ESWT) and ultrasonics (US) have been found to be effective for tennis elbow as highlighted in previously published randomized controlled trials (RCTs) and reviews. However, the efficacy of these two therapies in treating tennis elbow is unknown. This meta-analysis compares the effectiveness of ESWT and US in relieving pain and restoring the functions of tennis elbow following tendinopathy.

**Methods:**

RCTs published in the PubMed, Embase, Cochrane Library, and SpringerLink databases comparing ESWT and US in treating tennis elbow were identified by a software and manual search. The risk of bias and clinical relevance of the included studies were assessed. Publication bias was explored using funnel plot and statistical tests (Egger’s test and Begg’s test). The major outcomes of the studies were analyzed using the Review Manager 5.3.

**Results:**

Five RCTs comprising five patients were included in the present meta-analysis. The results revealed a significantly lower VAS score of pain in the ESWT group (1 month: MD = 4.47, *p* = 0.0001; 3 months: MD = 20.32, *p* < 0.00001; and 6 months: MD = 4.32, *p* < 0.0001) compared to US. Besides, the grip strength was markedly higher 3 months after the intervention in ESWT (MD = 8.87, *p* < 0.00001) than in the US group. Although no significant difference was observed in the scores of the elbow function after 3 months of treatment (SMD = 1.51, *p* = 0.13), the subjective scores of elbow functions were found to be better in the ESWT group (SMD = 3.34; *p* = 0.0008) compared to the US group.

**Conclusions:**

Although there was no significant difference in the elbow function evaluation scores between ESWT and US, the superiority of the ESWT group in the VAS of pain (both at 1 month, 3 months, and 6 months follow-ups) raised grip strength in ESWT group and the scores for subjective evaluation of efficacy indicated that ESWT offers more effective therapy for lateral epicondylitis than US therapy.

**Electronic supplementary material:**

The online version of this article (10.1186/s13018-019-1290-y) contains supplementary material, which is available to authorized users.

## Introduction

It is well known that lateral epicondylitis (LE), also known as tennis elbow, is one of the most ubiquitous cause of elbow pains among craftsmen [1–3]. It has an incidence of 1–3% in the general population and constitutes 7 per 1000 primary care consultations annually [4, 5]. Tennis elbow manifests as a tenderness over the lateral epicondyle of the humerus, as well as a pain on resisted dorsiflexion of the wrist [5, 6]. Although the cause of tennis elbow is often non-specific, it is most commonly associated with work-related or sports-related overuse of the elbow resulting in hypovascularity in the area [7–9]. Its symptoms can persist for half a year to 2 years, but may resolve naturally in a year or so [10, 11]. The pain mainly affects the dominant arm, and the severity of this condition tends to be high and persistent for longer duration in female [12].

Although the diagnosis of tennis elbow was standardized many years ago based on its symptoms [5, 13–15], treatments remain largely non-definitive and variable. There are, however, many therapies with beneficial effects, as revealed in some clinical studies [2, 3, 16]. Topical non-steroidal anti-inflammatory drugs (NSAIDs) are mainly prescribed for short-term pain relief, whereas oral NSAIDs are aimed at short-term improvement in pain relief and function. An extensor fasciotomy was demonstrated to be an effective treatment for refractory chronic lateral epicondylitis [17]. Extracorporeal shock wave therapy (ESWT), ultrasonic therapy (US), corticosteroid injections, physiotherapy, and acupuncture are effective therapies for long-term pain relief and/or functional improvement for patients with tennis elbow [18]. Due to their non-invasive and convenient nature, ESWT and US are considered important adjuvant interventions for treating tennis elbow [19].

Indeed, either of these two treatments is routinely used as the main adjuvant therapy [20, 21]. However, there is no consensus as to which method is the more effective in treating tennis elbow [22]. It is therefore meaningful to make a direct comparison between ESWT and US on their efficacy and safety. This study compares the therapeutic effects of these two therapies in reducing pain intensity, improving mobility in daily activities and self-evaluation of recovery.

## Materials and methods

### Searches

This study was conducted according to the guidelines outlined in the PRISMA (Preferred Reporting Items for Systematic Review and Meta-Analysis). The following databases were explored to extract relevant data: The Cochrane Library, PubMed, MEDLINE, SpringerLink, and OVID. Appropriate randomized controlled trials (RCTs) published between January 2001 and March 2019 were enrolled in this study. The following subject terms were employed in the literature search: tennis elbow, lateral epicondylitis, ultrasonics, and extracorporeal shock wave, and the entry terms related to the subject terms mentioned above were applied in the same way. The specific search employed was as follows: (((((ultrasonic) AND ultrasonics)) AND (((((((((((((((((extracorporeal shockwave therapies) or shockwave therapies, extracorporeal) or shockwave therapy, extracorporeal) or therapy, extracorporeal shockwave) or shock wave therapy) or shock wave therapies) or therapy, shock wave) or extracorporeal shock wave therapy) or extracorporeal high-intensity focused ultrasound therapy) or extracorporeal high intensity focused ultrasound therapy) or hifu therapy) or hifu therapies) OR therapy, hifu) or high-intensity focused ultrasound therapy) or high intensity focused ultrasound therapy)) or extracorporeal shock wave)) and ((tennis elbow) and (((((((((((((elbow, tennis) or elbows, tennis) or tennis elbows) or lateral epicondylitis) or epicondylitiden, lateral) or epicondylitis, lateral) or lateral epicondylitis) or epicondylitis, lateral humeral) or epicondylitiden, lateral humeral) or humeral epicondylitis, lateral) or humeral epicondylitis, lateral) or lateral humeral epicondylitis) or lateral humeral epicondylitis))) and ((randomized controlled trial [Publication Type] or (randomized [Title/Abstract] and controlled [Title/Abstract] and trial [Title/Abstract]))).

### Study inclusion and exclusion criteria

The titles and abstracts of the articles that appeared in the literature search were reviewed independently by two authors to evaluate their eligibility for enrollment. The authors settled any disagreements through discussion. A third person acted as a referee to adjudicate the debate between the investigators. The following criteria were used to select the articles: (1) clinical study was designed and conducted as a randomized controlled trials (RCTs), (2) the study made a comparison between ESWT and US on efficacy for treating lateral epicondylitis, (3) the article was written in the English language or had been translated into English, and (4) the major outcomes measured were the efficacy of pain relief and functional restoration. The exclusion criteria were as follows: (1) the study shared the same data set, (2) the evaluation methods did not address the major outcome, and (3) the participants included in the study had co-morbidities and/or other joint diseases such as hypertension and rheumatoid arthritis.

### Data extraction strategy

Based on the pre-determined criteria, the following data was extracted independently from the selected articles by the two authors: background information such as the country of the study, interventions, and major and minor outcomes, and the characteristics of the study subjects such as ethnicity, age, gender, and duration of the symptoms. A third investigator examined the discrepancies in data extraction. The results are provided in Additional file 1.

### Quality and risk of bias assessments

The modified Jadad scale was used to evaluate the quality of the studies, while the Cochrane Handbook for Reviews of Interventions (RevMan version 5.3) was used to assess the risk of bias. The enrolled articles were reviewed by two authors. Each of the studies was assigned a score corresponding to “low,” “high,” or “unclear” according to the following items: selection bias, performance bias, detection bias, attrition bias, reporting bias, and other biases. Any disagreements between the authors were resolved through discussion.

### Data synthesis and presentation

The RevMan statistical software (RevMan version 5.3) was used to analyze the data extracted from the enrolled articles. In this study, binary data was analyzed to provide a statistical summary of the risk ratios (RR) and the associated 95% confidence intervals (CI) (α = 0.05 for the inspection standards). The continuous data were expressed as means and standard deviations (SD), which were then pooled to a weighted mean difference (WMD) and 95% confidence interval (CI). Heterogeneity was examined by the *I*^2^ statistic. Outcomes with an *I*^2^ statistic of 25–50% were considered to have a low heterogeneity, and 50–75% a moderate heterogeneity, while *I*^2^ > 75% reflected high heterogeneity. For the outcomes with the *I*^2^ statistic exceeding 50%, subgroup analyses were conducted to investigate the sources of heterogeneity. A statistical significance was indicated by a *p* value < 0.05. The fixed effects were employed, for a greater statistical power.

## Results

### Literature search and study characteristics

**Fig. 1:**
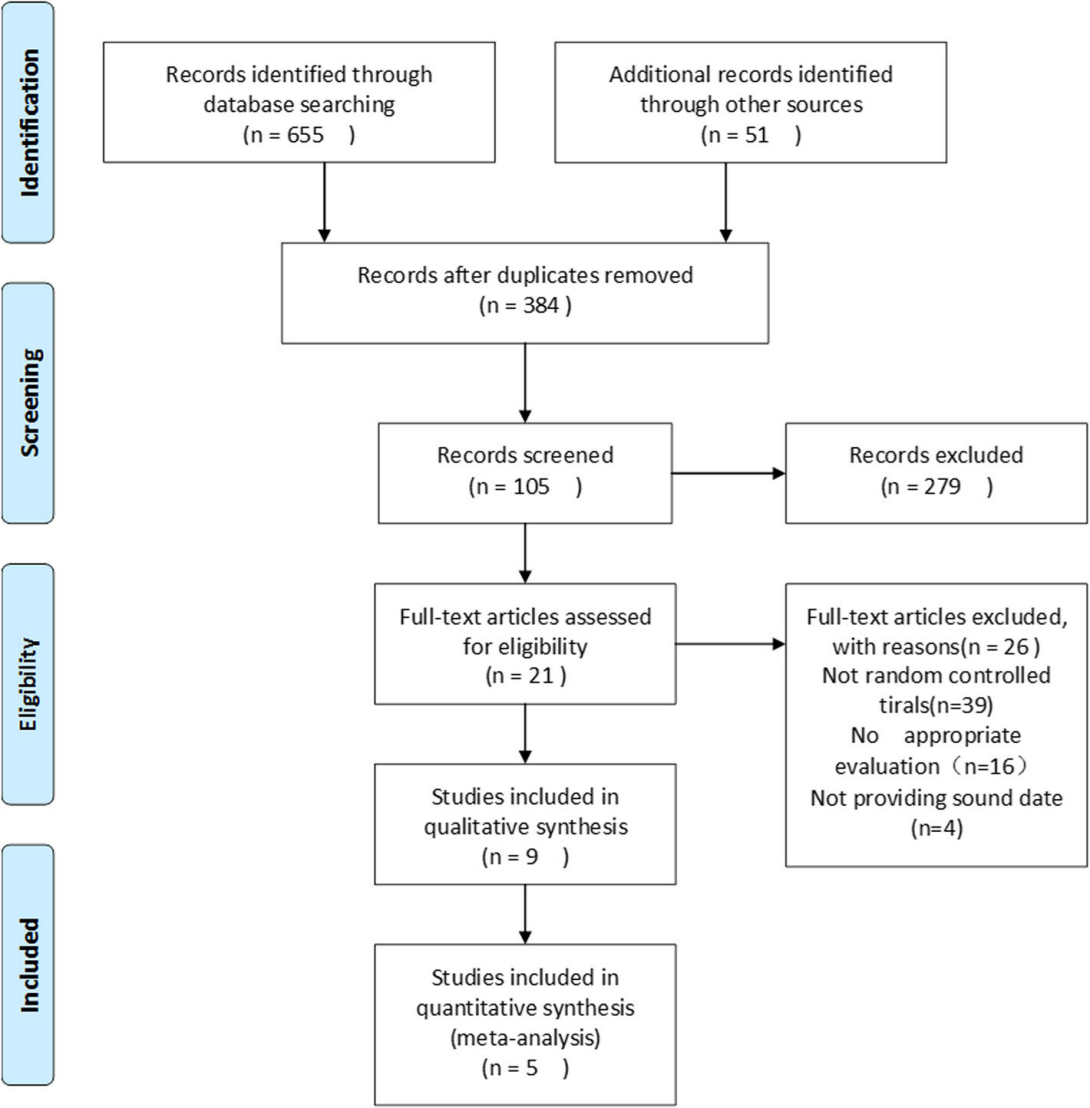
Flow diagram for the included studies

The literature search yielded 706 articles which were considered as potential studies. Three hundred eighty-four publications remained after removal of the duplicates based on the title and abstracts. After the preliminary screening of the 384 studies, a total of 21 manuscripts were further evaluated comprehensively. Finally, 5 articles were found to be eligible for the present meta-analysis. A total of 115 patients were enrolled in the ESWT group, while 118 patients were enrolled in the US group. Tables 1 and 2 summarize the demographic characteristics of the study subjects and quality scores of the studies. Figure 1 shows the literature selection process as described above (see Additional file 2). This study followed the PRISMA 2009 checklist as provided in Additional file 3.

**Table 1:** The characteristics of included studies

Study	Year	Country	Patients (*n*)		Age (year)		Gender				Symptom duration (months)		Side of involvement				Study design
			ESWT	US	ESWT	US	ESWT		US		ESWT	US	ESWT		US		
							Male	Female	Male	Female			Left	Right	Left	Right	
Gunduz	2012	Turkey	20	19	44.9 ± 9.9	43.6 ± 9.1	8	12	5	14	30 (1–90)	30 (7–90)	5	15	4	15	RCT
Kubot	2017	Poland	30	30	47.6 ± 7.66	43.9 ± 9.44	8	22	17	13	> 2	> 2	–	–	–	–	RCT
Pawel	2015	Poland	25	25	47.9 ± 4.4	49.0 ± 4.5	–	–	–	–	14.9 ± 2.1	15.1 ± 1.9	0	25	0	25	RCT
Yalvac	2018	Turkey	20	24	43.75 ± 4.52	46.04 ± 9.24	5	15	8	16	7.9 ± 3.3	8.2 ± 3.6	3	17	4	20	RCT
Soheir	2013	Egypt	20	20	38.4 ± 3.67	38.25 ± 4.19	12	8	12	8	>10	>10	15	4	17	4	RCT

**Table 2:** Characteristics of the four trials selected showing general intervention information

Study	Follow month	Interventions		Outcome measures	Distribution kit	Calculation software
		ESWT	US			
Gunduz	6	ESWT (pressure 1.4 bar, frequency 4.0 Hz, number 500) for ten sessions	Ultrasound therapy (1 W/cm^2^, 5 min) and friction massage (5 min) for ten sessions	VAS, grip strength, pinch strength	Sealed numbered envelopes without strata	Statistical Package for Social Sciences, version 11.5
Kubot	6	First phase: 2000 pulses with a wavelength of 8 Hz and a pressure of 1.5–2.5 bar; second: 2000 pulses at 8 Hz and 2.5–3.5 bar	First phase 3 min, a 5 cm^2^ with 1 MHz, producing a wave of 0.5 W/cm^2^; second lasting 2 min, same as before	VAS, subjective pain, frequency f pain, use of analgesic drugs. Mobility limitation of the affected limb	Randomized by unspecific way	SPSS 24 program
Pawel	3	1000, 1500, and 2000 pulses, pressure, 2.5 bar; frequency, 8 Hz; energy density, 0.4 mJ/mm^2^	Intensity, 0.8 W/cm^2^; 100% fill; carrier frequency, 1 MHz. Ten treatments 3 times per week	VAS (rest, grip, palpation, Thomsen test, Chair test); overall outcome score	MedCalc statistical software	MedCalc statistical software version 15.2.1
Yalvac	3	10e15 Hz, 1.5e2.5 bar energy density, 2000 pulses, once a week for three sessions	1 cm^2^ application area, at 1.5 W/cm^2^, 1 MHz frequency, continuous mode in painful area, 5 min once a day, 5 days a week, for 10 sessions in total	VAS, grip strength evaluation, pressure-pain, DASH/quickDASH, short Form-36	Coin tossing method	Number Cruncher Statistical System (NCSS)
Soheir	6	Orthospec (Medispec LTD, Germantown, MD) portable ESWT device; single phase 60/50 Hz and 10/5A	Ultrasound device Phyaction 190 serial number 2745, 230 V, 300 mA/50–60 Hz, Pus: 8w. Continuous mode 1.5 w/cm, frequency 1 MHz	VAS at ease/work, grip strength, Chair test, Cozen test, tennis albow test	The use of computer-based 1:1 randomization scheme	Computer program SPSS 16

### Risk of bias in included studies

**Fig. 2:**
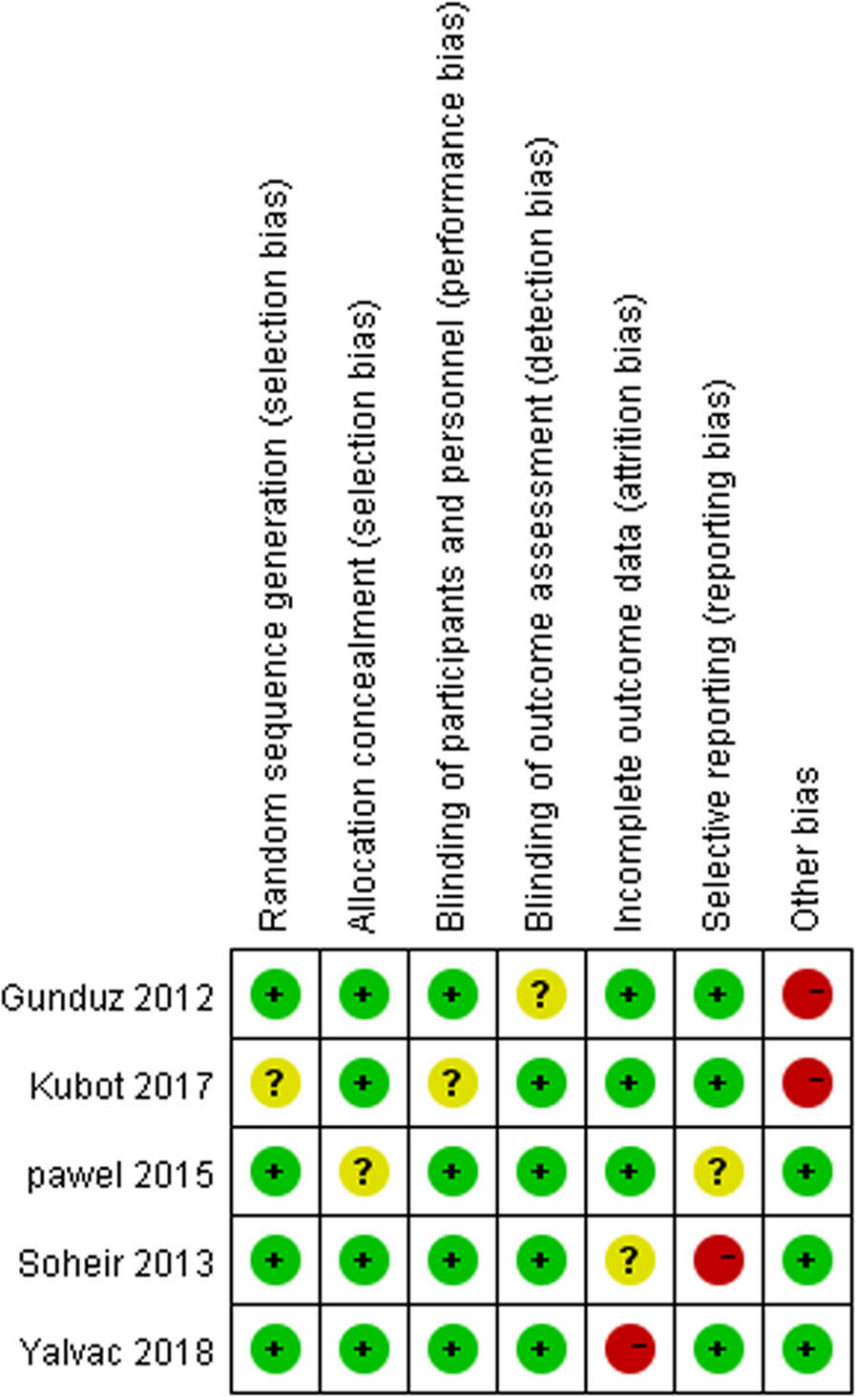
Risk of bias summary of the included studies

**Fig. 3:**
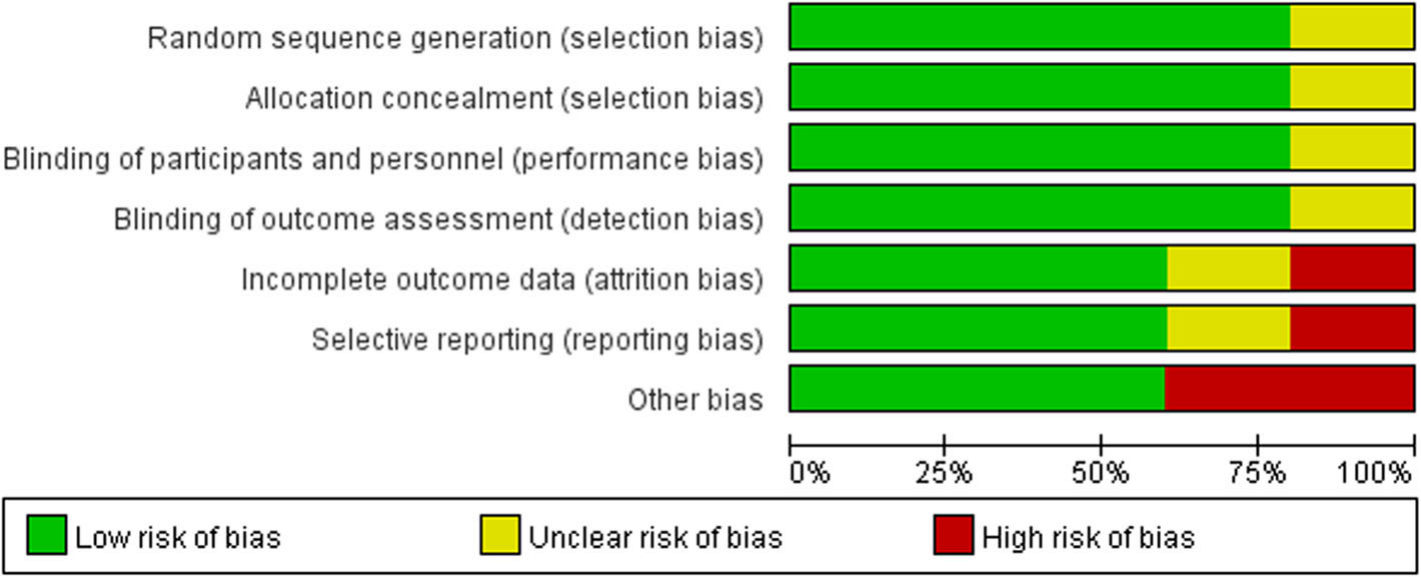
Risk of bias summary

The risk of bias for each of the assessed studies and the results are summarized in (Fig. 2 and Fig. 3). While specific methods for random sequence generation were not mentioned in two trials [23, 24], all of the selected studies claimed a randomized trial design. One trial [25] did not explain their methods for allocation concealment. Blinding processes were not clearly described in two studies [25, 26]. The one remaining trial [27] was considered to have a high risk due to inadequate blinding.

### Pain scores

**Fig. 4:**
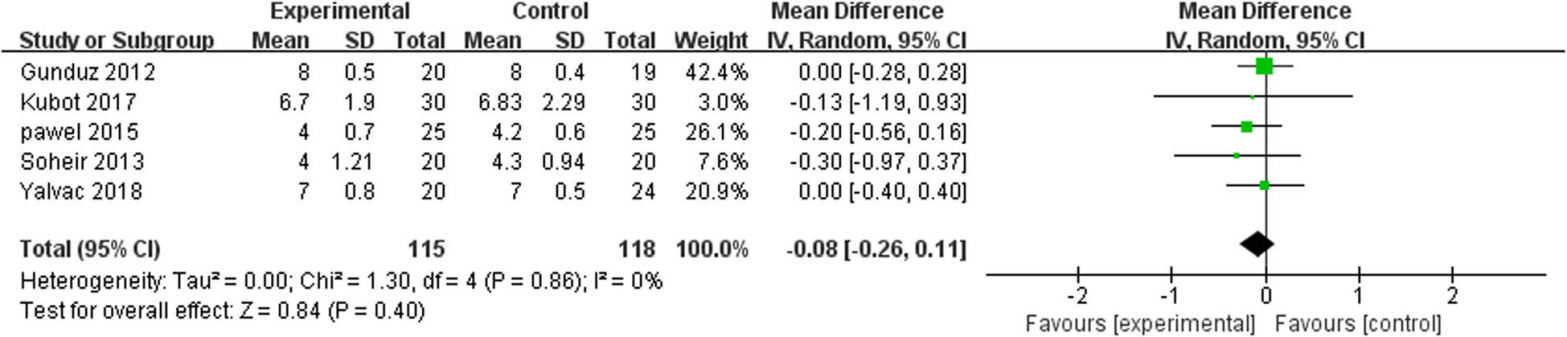
Forest plot of pain score when comparing ESWT group with US group before treatment

**Fig. 5:**
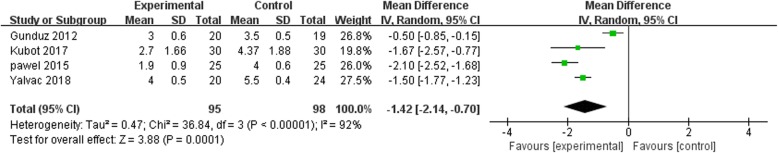
Forest plot of pain score when comparing ESWT group with US group at 1 month after treatment

**Fig. 6:**
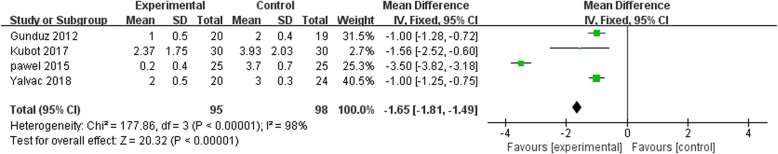
Forest plot of pain score when comparing ESWT group with US group at 3 months after treatment

**Fig. 7:**
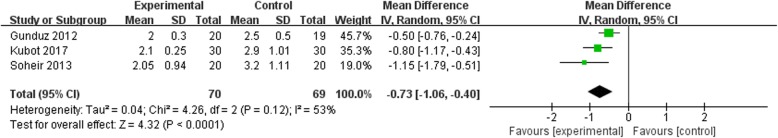
Forest plot of pain score when comparing ESWT group with US group at 6 months after treatment

The visual analogue scale (VAS) was adopted by all five [23–26, 36] trials to evaluate the degree of pain relief. As shown in Fig. 4, there were no differences in the pain caused by tennis elbow between the treatment groups before the intervention (MD = 0.84, *p* = 0.54, *I*^2^ = 0%). However, the ESWT group showed a significantly large reduction in the level of pain after the treatment at 1 month follow-up (MD = 4.47, *p* = 0.0001, *I*^2^ = 92%) (Fig. 5) while the difference in the pain relief between the treatment groups persisted at 3 months follow-up (MD = 20.32, *p* < 0.00001, *I*^2^ = 98%) in four [23–26] trials (Fig. 6). On the other hand, a remarkable difference (MD = 4.72, *p* = 0.0001, *I*^2^ = 53%) in VAS score existed at 6 months between ESWT group and US group (Fig. 7) in three [23, 24, 36] trials. These results suggest that the ESWT has a superior efficacy than the US in eliminating the pain in both short- and long-term.

**Fig. 8:**
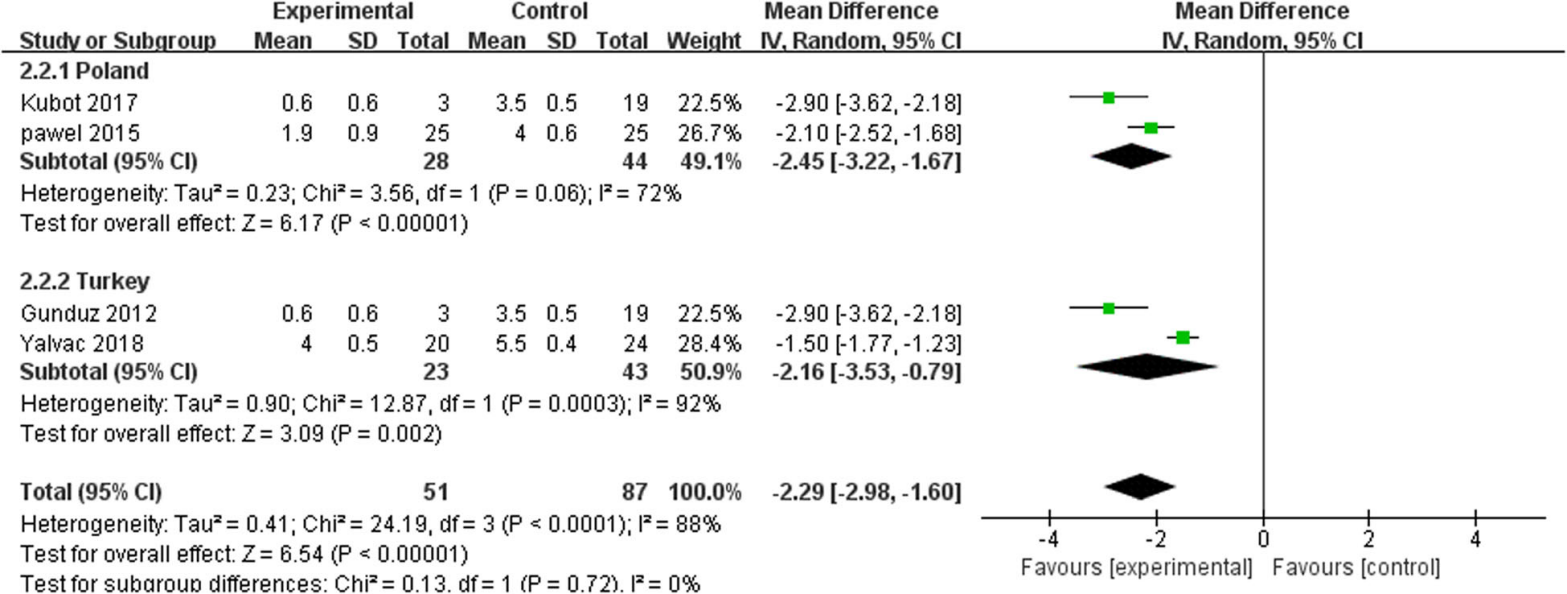
Forest plot of subgroup analysis in pain score when comparing ESWT group with US group with 1 month after treatment

**Fig. 9:**
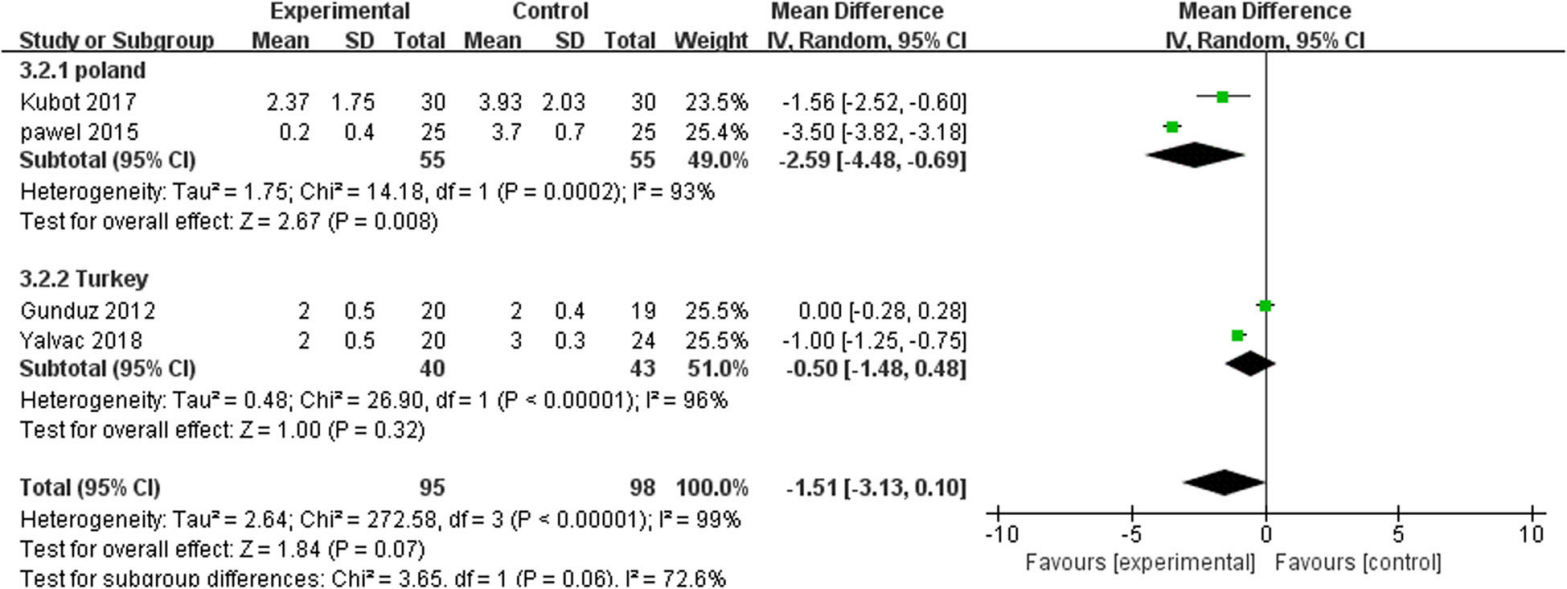
Forest plot of subgroup analysis in pain score when comparing ESWT group with US group in 3 months after treatment

A subgroup analysis based on the race of the subjects was conducted to explore the sources of the high heterogeneity observed in the pain scores across the studies. The results of subgroup analysis for the Polish subgroup (MD = 6.17, *p* < 0.00001, *I*^2^ = 72%) and Turkish subgroup (MD = 3.09, *p* = 0.002, *I*^2^ = 92%), as well as the total effect (MD = 6.54, *p* < 0.0001, *I*^2^ = 88%) at 1 month follow-up are shown in Fig. 8. The results for the Polish subgroup (MD = 2.67, *p* = 0.008, *I*^2^ = 93%), Turkish subgroup (MD = 1.00, *p* = 0.32, *I*^2^ = 96%), and total effect (MD = 1.84, *p* = 0.07, *I*^2^ = 99%) at 3 months follow-up are summarized in Fig. 9. The results of the subgroup analysis are explained in the “Discussion” section.

### Grip strength at 6 months after intervention

**Fig. 10:**
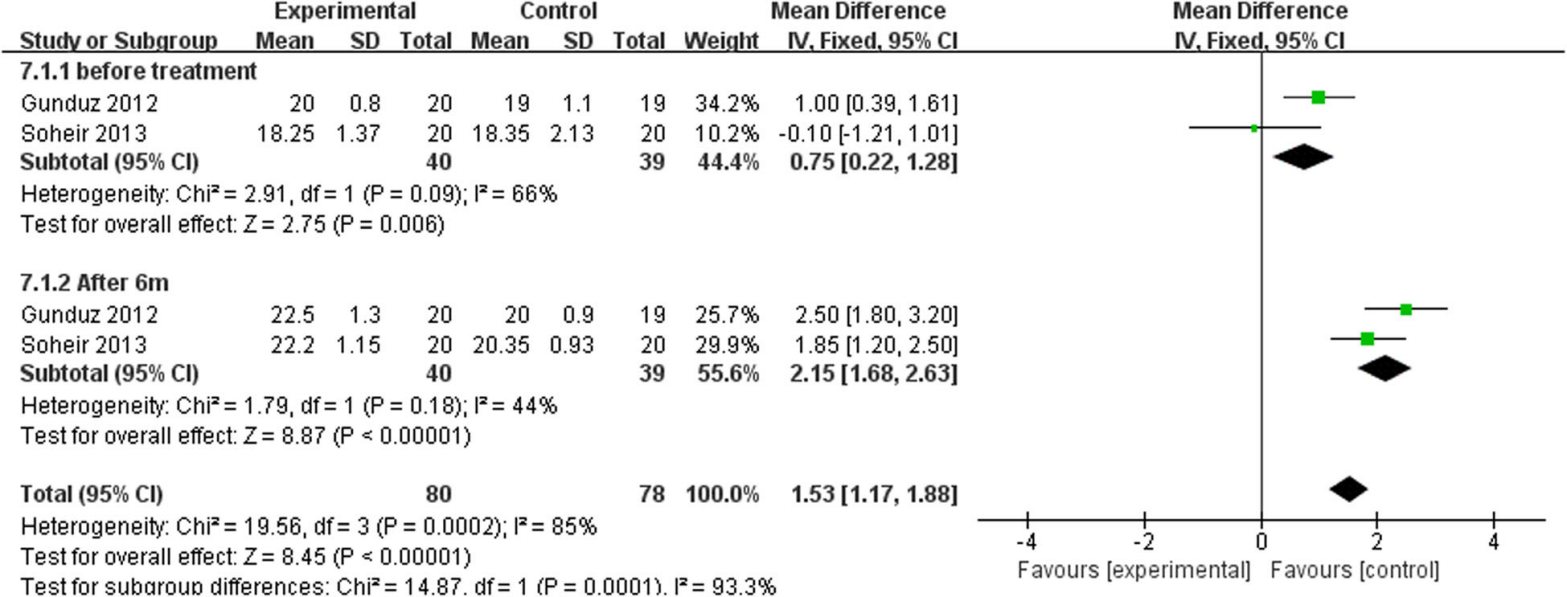
Forest plot of grip strength evaluation when comparing ESWT group with US group at 1 month and 6 months after treatment

Two [23, 36] of the five trials were enrolled to prepare evaluation of grip strength at 6 months after intervention. A comparison was made between ESWT group and US group at 1 and 6 months (Fig. 10). The results revealed that the ESWT group had a better recovery of grip strength compared to the US group (MD = 2.75, *p* = 0.06, *I*^2^ = 66%) 1 month after the treatment. Meanwhile, the difference in comparison between ESWT and US group at 6 months after therapy revealed the same outcome (MD = 8.87, *p* < 0.00001, *I*^2^ = 44%). For these reasons, ESWT resulted in better recovery of grip strength in LE patients than US therapy in the long and short run.

### Evaluation of the elbow functions

**Fig. 11:**
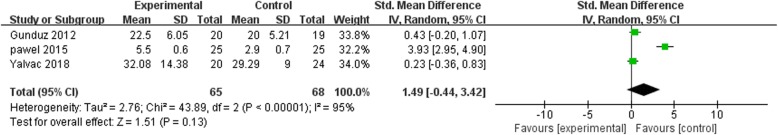
Forest plot of evaluation scores of elbow function when comparing ESWT group with US group at 3 months after treatment

Three of the five trials [23, 25, 26] made a comparative evaluation of the elbow functions at follow-ups varying from 1 to 6 months. Elbow function evaluation covered the range of motion of joints, muscle strength, pain, and activities of daily living. Some special function evaluation items included the Chair test, Thomas test, and tennis elbow test. The common time point for follow-up in these trials was 3 months after the treatment (Fig. 11). There were no significant differences in the function scores between the treatment groups at 3 months follow-up (SMD = 1.51, *p* = 0.13, *I*^2^ = 95%), indicating that ESWT and US have similar effects on the functional improvement.

### Subjective evaluation of therapy efficacy

**Fig. 12:**
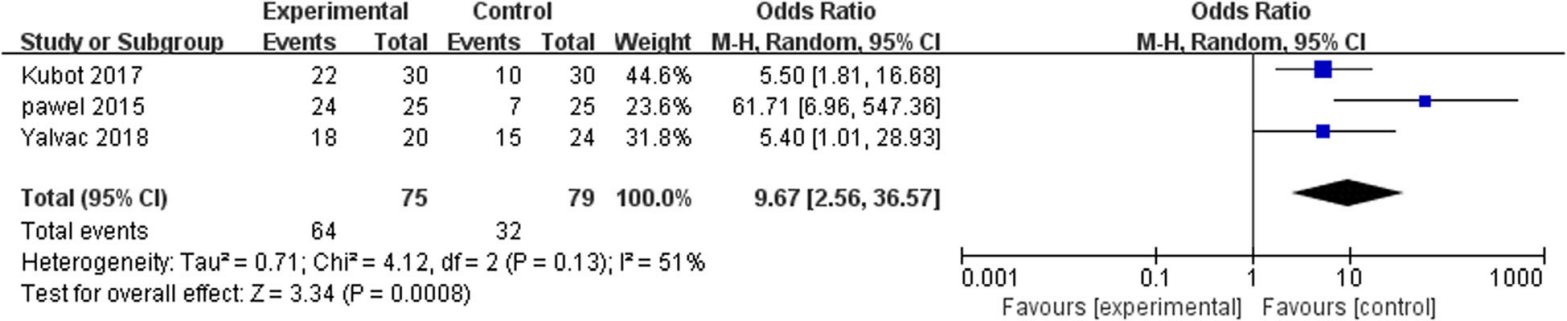
Forest plot of judgment of comprehensive efficacy when comparing ESWT group with US group at 3 months after treatment

Of the four studies, three trials [24–26] performed a subjective evaluation of efficacy in pain relief, restoration of the elbow functions, impact on subjects’ ability to work, and so on. There was a significant difference (SMD = 3.34, *p* = 0.0008, *I*^2^ = 51%) between the ESWT and US groups, as shown in Fig. 12; thus, ESWT provided more efficacy in treatment than the US.

## Discussion

Tennis elbow is a common chronic joint condition, which is characterized by pain and tenderness over the elbow [5, 6]. Limited movements at the elbow joint may severely disrupt daily activities and work, resulting in economic burden to the society [27]. According to previous studies [2, 3, 16], there are many therapies for tennis elbow, including topical and oral NSAIDs, corticosteroid injections, ultrasonics (US), and extracorporeal shock wave therapy (ESWT). Due to the non-invasive nature and little to no side effects or adverse events associated with ESWT and US, they are preferred by many patients and clinicians as the main adjuvant therapies for tennis elbow [19]. In addition, the efficacy of these two therapies has been supported by a growing number of clinical studies [28–31]. In most cases, either the US or the ESWT is elected per case because of their similar functionality. In spite of that, there is no consensus as to which therapeutic approach is superior in efficacy [22]. To address this question, the current study compares the efficacy of ESWT and US in the treatment of tennis elbow.

Studies that met the criteria for meta-analysis were examined to extract data on pain relief and functional improvement following ESWT or US treatment in subjects suffering from tennis elbow. In addition, the risk of bias in each of the studies was evaluated via the modified Jadad scale. In this meta-analysis, it was observed that ESWT group had a higher reduction in pain after 1 month of treatment as compared to the US group (MD = 3.88, *p* = 0.0001, *I*^2^ = 92%); similar results were obtained after 3 months (MD = 20.32, *p* < 0.00001, *I*^2^ = 98%) of treatment. These results indicate that ESWT is a superior therapy compared with US in providing a long-term pain relief in tennis elbow. More importantly, these results were consistent with the previous findings by Rompe et al. [28–31]. However, there was high heterogeneity in the pain scores results. Thus, a subgroup analysis was conducted by dividing the short-term and intermediate-term pain scores based on race. It was found that the Turkish subgroup had a higher heterogeneity (MD = 3.09, *p* = 0.002, *I*^2^ = 92%) than the Polish subgroup (MD = 6.17, *p* < 0.00001, *I*^2^ = 72%) at 1 month follow-up, and the same relationship was true at 3 months follow-up for the Polish subgroup (MD = 2.67, *p* = 0.008, *I*^2^ = 93%) and Turkish subgroup (MD = 1.00, *p* = 0.32, *I*^2^ = 96%). However, the study by Smidt et al. [32] revealed that there is a uniform course of recovery for tennis elbow without a clinical heterogeneity, suggesting that a high heterogeneity attributable to the ethnic variation may not be clinically relevant.

In term of the functional recovery, there was no significant difference in the functional scores when the two treatment groups (SMD = 1.51, *p* = 0.13, *I*^2^ = 95%) were compared, as summarized in Fig. 8**.** This outcome of function evaluation was different from the others because of the following reasons. The content of function evaluation consisted of varied parts from trial to trial and could be accounted for. Besides, it was difficult to perform assessment quantitatively and in a timely manner due to the complexity of certain operation. Further, the diversity of function assess scores would offset some meaningful differences internally caused by therapies, which exhibited an ultimate smooth trend. Taking these two points into consideration, we did not pay much attention into the different consequence from function evaluation section and the result should also be treated cautiously. Further, a significant difference was observed in the average scores between the ESWT group and US group (SMD = 3.34, *p* = 0.0008, *I*^2^ = 51%) after subjective evaluation of efficacy. Therefore, it can be concluded that ESWT is more effective in enhancing recovery from tennis elbow compared to US. A moderate heterogeneity was noted among the studies. A subsequent subgroup analysis revealed that variations in the judgment scores adopted by these trials partly accounted for the observed heterogeneity, for which SMD was used to offset some of the effects.

The higher efficacy of ESWT in pain relief and subjective improvements in tennis elbow may be explained by two mechanisms. Firstly, the energy released by ESWT is greater than that of ultrasonic wave. This would likely enable it to better stimulate pain receptors located in the skin, muscle, connective tissue, bone, and joint, as well as to activate unmyelinated C fibers and A delta fibers to initiate the “gated” pain control system, leading to an analgesic effect [33]. Secondly, ESWT causes a large number of tiny bubbles created within tissues, which rapidly expand and burst under the action of shock wave, resulting in high-speed liquid micro-jet and impact effect. This cavitation effect is particularly effective for re-opening occluded microvessels and releasing the soft tissue adhesion at joints [34].

The present study has some shortcomings which need to be highlighted. Firstly, the number of enrolled trials is small which limits the generalizability and contingency of the results. Secondly, the side effects of ESWT and US, such as temporary reddening of the skin, pain, and formation of small hematomas, were not evaluated during follow-up, which differs from the study by Haake et al. [35]. The high heterogeneity among the results weakens the reliability of the results. Therefore, a longer study duration is needed to assess the efficacy of ESWT and US on the tennis elbow function and to explore the optimal therapeutic setting of ESWT.

Nevertheless, the results from this meta-analysis indicate that the efficacy of ESWT is superior to that of US in terms of pain relief and overall recovery in tennis elbow.

## Conclusions

This meta-analysis reveals that ESWT effectively relieves tennis elbow pain at 1 month and 3 months follow-ups compared to US. The subjective evaluation of efficacy showed that ESWT group was superior to US group, although no significant difference was observed in the elbow function scores between the two groups. Together, these results lead to the conclusion that ESWT is a superior adjuvant therapy for tennis elbow compared to US.

## Additional files

Additional file 1: Data extraction excel. (XLSX 22 kb)

Additional file 2: PRISMA 2009 flow diagram word version. (DOC 34 kb)

Additional file 3: PRISMA 2009 checklist. (DOC 62 kb)

## Data Availability

We state that the data will not be shared because all the raw data are present in the figures included in the article.

## Abbreviations

ESWT: Extracorporeal shock wave
LE: Lateral epicondylitis
RCTs: Randomized controlled trials
US: Ultrasonic
VAS: Visual analogue scale/score
