# Supplementary material for: A comparative study of the efficacy of ultrasonics and extracorporeal shock wave in the treatment of tennis elbow: a meta-analysis of randomized controlled trials
Source: J Orthop Surg Res. 2019 Aug 6;14:248. doi: 10.1186/s13018-019-1290-y (PMC6683364; doi:10.1186/s13018-019-1290-y)
Supplement: Supplementary file 2 — PRISMA 2009 flow diagram word version. (DOC 34 kb) [file 13018_2019_1290_MOESM2_ESM.doc]

**Screening**

**Included**

**Eligibility**

**Identification**

Records identified through database searching
(n = 655 )

Additional records identified through other sources
(n = 51 )

Records after duplicates removed
(n = 384 )

Records screened
(n = 105 )

Records excluded
(n = 279 )

Full-text articles assessed for eligibility
(n = 21 )

Full-text articles excluded, with reasons(n = 26 )

Not random controlled tirals(n=39)

No appropriate evaluation（n=16）

Not providing sound date

(n=4)

Studies included in qualitative synthesis
(n = 9 )

Studies included in quantitative synthesis (meta-analysis)
(n = 5 )

Fig. 1 Flow diagram for the included studies
